# The Creation of the CAPES Journals Portal, aka Portal Periódicos: A Personal Recollection

**DOI:** 10.1021/acsomega.5c11518

**Published:** 2025-12-22

**Authors:** Luiz Valcov Loureiro

**Affiliations:** † Department of Chemical Engineering, 28133 University of São Paulo, 05508-010 São Paulo, São Paulo, Brazil; ‡ Fulbright Comission Brazil, 70701-000 Brasília, Distrito Federal, Brazil

## Abstract

This viewpoint describes the trajectory of the creation and consolidation of the CAPES Journals Portal (Portal Periódicos), a pioneering initiative that transformed access to scientific information in Brazil. Conceived in the late 1990s within the Brazilian Federal Agency for Support and Evaluation of Graduate Education (CAPES), the Portal emerged from the need to democratize access to knowledge in a country of continental dimensions, where printed journals were scarce, costly, and unevenly distributed. By replacing paper-based subscriptions with a centralized, digital library model, CAPES fostered nationwide access to world-class scientific publications. This viewpoint recounts the political, financial, and technological challenges faced during its implementation, the strategic inclusion of São Paulo institutions to ensure long-term sustainability, and the enduring impact of the Portal as an inclusive infrastructure for research and graduate education in Brazil. Twenty-five years later, the CAPES Journals Portal stands as a testament to visionary public policy, serving as the backbone of Brazilian science and a model of equitable access to knowledge.

## Graphical abstract

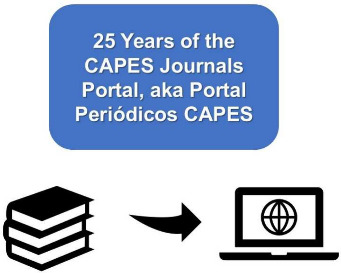

When I joined the Brazilian Federal Agency for Support and Evaluation of Graduate Education (CAPES) at the Ministry of Education in the mid-1990s as Director of Programs, Brazilian science faced a crucial challenge: limited access to knowledge in a developing country larger in size than the continental United States. Back then, research libraries across the country relied on printed journals, acquired primarily with CAPES funds, that were expensive, slow to arrive, and very unevenly distributed. Only a few great universities—the University of São Paulo (USP), the Federal University of Rio de Janeiro (UFRJ), and the University of Campinas (UNICAMP)—had decent in-house collections, while smaller institutions depended on interlibrary loans and the “COMUT” system to request photocopies of journal articles from a Brazilian institution with a subscription to the journal of interest. It was a time when information traveled by mail and “COMUT” turn-around times were often of the order of 1 to 2 months. Moreover, budgets were tight, especially with the fluctuations of the U.S. dollar that directly affected the cost of subscriptions.

By the late 1990s, Internet access to scientific journals was beginning to emerge, primarily through expensive paid subscriptions. Discussions about open access also started, leading to the Budapest Declaration (2002). In Brazil, the São Paulo Research Foundation (FAPESP) had begun to license a few electronic reference databases like the Web of Science exclusively to the universities in the state of São Paulo. I remember thinking that this new model—centralized, online, and instantly accessible—was the way forward. Yet, we faced multiple obstacles: insufficient funding, limited Internet bandwidth, and a cultural attachment to paper. Many librarians and even researchers distrusted the digital medium, questioning its reliability and permanence.

Still, it was clear to me that democratizing access to scientific information was essential for Brazil’s development, so in 1999, we decided to take a bold step: stop buying paper-based journals and focus on building a national digital library of scientific journals—the CAPES Journals Portal, aka Portal Periódicos. This virtual library would bring together all major universities and research institutes under one virtual roof, providing any researcher, professor, or student in the country with access to world-class publications via their home institution.

The first negotiations were arduous. We dealt with powerful publishing houses—Elsevier and Springer—and scientific associations—ACS, IEEE, and others—and each contract involved complex and costly arrangements at a time when a currency crisis made every dollar precious. Parallel to this, we strengthened the Internet infrastructure of the Brazilian National Research Network (RNP) to ensure that it could handle the anticipated increased digital access. Without better Internet connectivity, the Portal Periódicos would have been useless.

In 2001, after months of technical adjustments and persuasion campaigns, the Portal Periódicos went online. Convincing librarians and researchers required patience and diplomacy. We had to show that digital access was not the enemy of the printed word but rather an evolution of libraries themselves. Working almost day and night with a small, highly motivated team—people who believed, as I did, that we were building something transformative—was exhausting but profoundly rewarding.

As the end of 2001 approached, with a newly elected president and administration set to take office in January 2002, there were serious concerns about the continuity of the Portal Periódicos. Brazil does not differ from other countries in many respects. The sustainability of public policies—even highly successful ones—often depends less on their technical merit than on the strength of the constituencies that support them. Programs endure when their beneficiaries, especially those with the greatest capacity for political mobilization, make it clear that discontinuation would carry significant political costs for the government of the day, especially when one had to justify to government officials why it was a worthy investment in the future of Brazil to pay “more than a movie theater ticket” per article, as one government minister said at the time.

Up to that point, institutions in the state of São Paulo—the most scientifically productive research community in the country—had only partial access to the Portal Periódicos. This was because the state funding agency FAPESP already provided access to a number of leading scientific journals and reference databases through its budget. However, this arrangement left the Portal Periódicos politically vulnerable: without the active participation of São Paulo, the base of political support for the CAPES virtual library was weaker. Although it represented a major financial burden for CAPES, the inclusion of São Paulo was a strategic decision deemed essential for securing the program’s survival. Time has proven this decision to be right. By bringing in São Paulo, the Portal Periódicos became indispensable to the entire national academic system, ensuring that researchers across Brazil could work with the same tools and ready access to information, a legacy that continues to sustain Brazilian science today.

The Portal Periódicos’s early catalog included around a thousand titles and five major reference databases (Figure 1). Although that might seem modest by today’s standards, it represented a revolution at the time: suddenly, scholars from every region of Brazil could access the same scientific information as their peers, not only those at the best universities in São Paulo but also those at the world’s top universities. Over time, the Portal Periódicos has grown and diversified to include tens of thousands of journals in all fields of knowledge and has transformed into an indispensable foundation of graduate education and research in Brazil.

**Figure 1:**
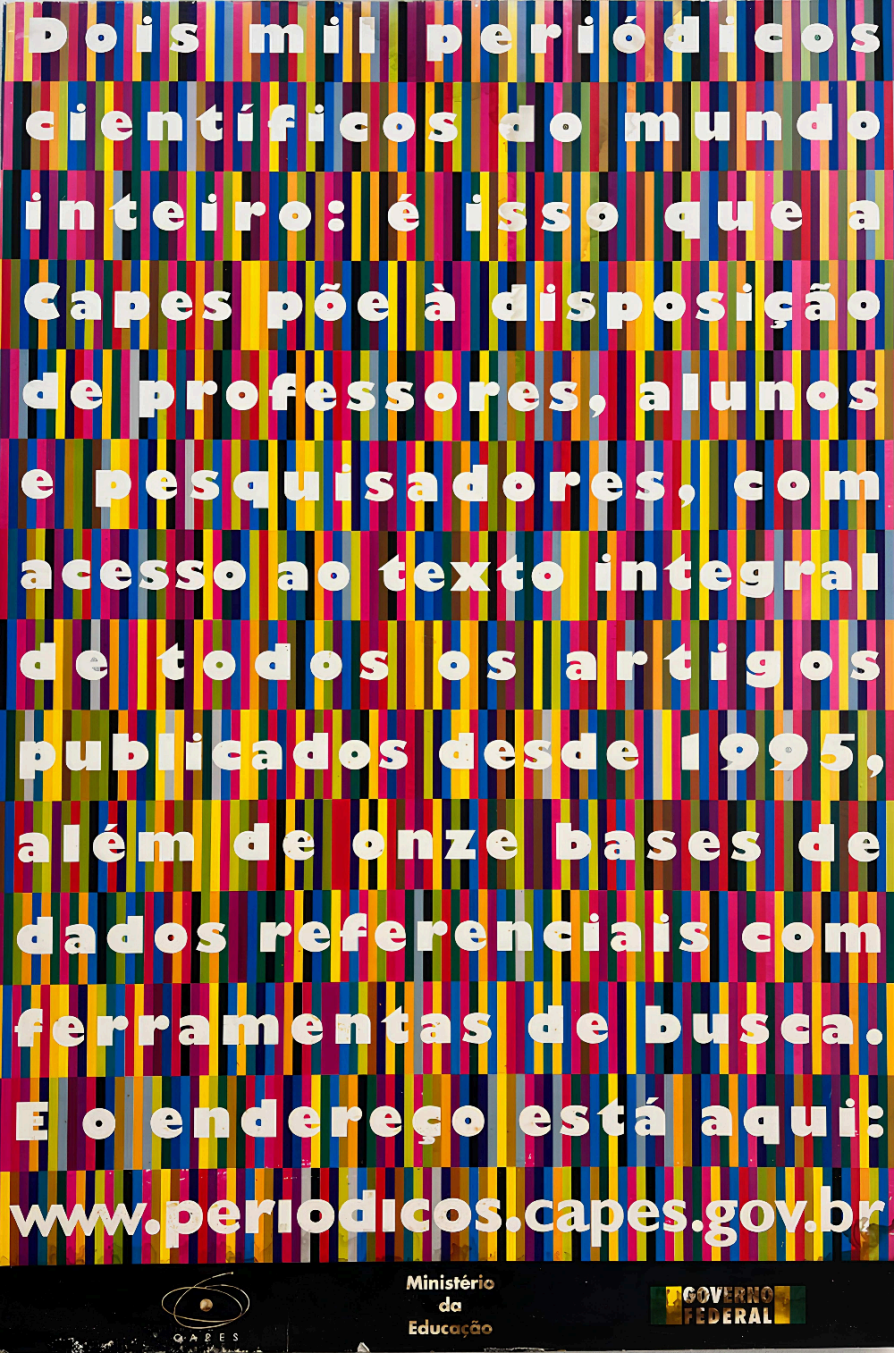
Commemorative board of the CAPES Journals Portal, aka Portal Periódicos, which in 2025 marks 25 years since its launch. On the board, it reads *Two thousand journals from all over the world —that is what CAPES makes available to professors, students, and researchers, providing full-text access to all articles published since 1995, as well as 11 reference databases with search tools. And here’s the address:* www.periodicos.capes.gov.br. The photograph was taken by the author. The CAPES and Portal de Periódicos logos are registered trademarks of Coordenação de Aperfeiçoamento de Pessoal de Nível Superior (CAPES) and are used here for academic and illustrative purposes only.

Looking back, I consider the creation of the Portal Periódicos during my tenure as Director of Programs at CAPES to be one of the most meaningful achievements of my professional life. It not only modernized academic infrastructure but also symbolized a profound act of inclusion. Instant access to knowledge that had been previously concentrated at only a few premier institutions became a shared public good equally available to all researchers throughout Brazil, from the Amazon to the beaches and interior of the northeastern states and down through the Pantanal and Minas Gerais to the southern frontiers with Argentina and Uruguay.

Today, 25 years later, the Portal Periódicos remains the lifeblood of Brazilian science and scholarly endeavor, the arterial system that interconnects our researchers with the global body of knowledge. It stands as proof that vision, collaboration, and persistence can overcome financial and technological barriers. Even if CAPES itself were to change someday, I am convinced that the Portal Periódicos would endure because it has become essential to who we are as a scientific nation.

